# Costly avoidance of Pavlovian fear stimuli and the temporal dynamics of its decision process

**DOI:** 10.1038/s41598-022-09931-1

**Published:** 2022-04-21

**Authors:** Juliane M. Boschet, Stefan Scherbaum, Andre Pittig

**Affiliations:** 1 Department of Psychology (Biological Psychology, Clinical Psychology, and Psychotherapy), University of Würzburg, Würzburg, Germany; 2 Department of Psychology, Technische Universität Dresden, Dresden, Germany; 3 Translational Psychotherapy, Department of Psychology, Friedrich-Alexander University Erlangen-Nürnberg (FAU), Erlangen, Germany

**Keywords:** Human behaviour, Emotion, Motivation

## Abstract

Conflicts between avoiding feared stimuli versus approaching them for competing rewards are essential for functional behavior and anxious psychopathology. Yet, little is known about the underlying decision process. We examined approach-avoidance decisions and their temporal dynamics when avoiding Pavlovian fear stimuli conflicted with gaining rewards. First, a formerly neutral stimulus (CS+) was repeatedly paired with an aversive stimulus (US) to establish Pavlovian fear. Another stimulus (CS−) was never paired with the US. A control group received neutral tones instead of aversive USs. Next, in each of 324 trials, participants chose between a CS−/low reward and a CS+/high reward option. For the latter, probability of CS+ presentation (Pavlovian fear information) and reward magnitude (reward information) varied. Computer mouse movements were tracked to capture the decision dynamics. Although no more USs occurred, pronounced and persistent costly avoidance of the Pavlovian fear CS+ was found. Time-continuous multiple regression of movement trajectories revealed a stronger and faster impact of Pavlovian fear compared to reward information during decision-making. The impact of fear information, but not reward information, modestly decreased across trials. These findings suggest a persistently stronger weighting of fear compared to reward information during approach-avoidance decisions, which may facilitate the development of pathological avoidance.

## Introduction

In daily life, stimuli and situations that are perceived as rewarding facilitate approach behavior, whereas those perceived as aversive or punishing facilitate avoidance^1,2^. In this context, anxiety disorders are assumed to be characterized by an imbalance of approach and avoidance tendencies during decision-making^3^ towards excessive avoidance behavior^4^. Oftentimes, fear-relevant situations (e.g., social gatherings in socially anxious individuals) are persistently avoided even if avoidance is linked to missing out on competing positive outcomes (e.g., positive social interactions). Severe impairments and the maintenance of fear and anxiety are common consequences^5–7^. Accordingly, a key therapeutic goal in the treatment of anxiety disorders is to reduce maladaptive avoidance (e.g., Ref.^8^). Research on how fear is acquired and how it shapes avoidance behavior is thus essential to inform and optimize treatment for anxiety disorders.

When examining avoidance behavior, two key factors need to be considered. First, it must be noted whether avoidance is linked to costs. In experimental conditioning research on fear and avoidance learning, avoidance is traditionally operationalized through pre-defined avoidance responses (e.g., button press) which prevent a single upcoming aversive stimulus (e.g., electrical stimulation). Such procedures efficiently capture low-cost avoidance (i.e., avoidance requires minimal costs or effort). However, it has been argued that their validity for costly avoidance in clinical settings is limited^6,9^. In support, we recently demonstrated that patients with anxiety disorders compared to matched healthy controls show similar levels of low-cost avoidance, but fail to reduce avoidance in presence of competing rewards for approach^10^. Thus, more and more studies apply approach-avoidance conflict tasks where approach is concurrently linked to varying aversive but also appetitive outcomes (e.g., monetary incentives). Choosing to avoid is therefore associated with safety but also costs, paralleling the impairments caused by pathological avoidance^5,6,9^. Results show that, despite competing rewards, avoidance increases notably with increasing likelihood of aversive consequences^11–15^. But importantly, avoidance behavior is reduced when rewards for approach are high^11–13^. These findings highlight that avoidance is not solely determined by potential threat, but also susceptible to other factors such as competing rewards for approach, social demand, trait anxiety, and stress (for an overview, see Ref.^6^).

Second, an important distinction must be made regarding the stimuli to which avoidance is directed. To date, most research on fear and avoidance learning focuses on avoidance of innately aversive unconditioned stimuli (i.e., US-avoidance^6^). Since USs, such as painful stimuli, indicate potential physical or mental harm^16^, US-avoidance is, in principle, an adaptive response. Clinical avoidance behavior, in contrast, is typically disproportionate, rigid, and often directed at stimuli associated with a perceived but not necessarily objective threat despite tremendous costs and impairments (e.g., public transport in patients with agoraphobia)^4^. Pavlovian fear learning, i.e., learning that a certain stimulus predicts an aversive event, is a model for the acquisition of fear towards such stimuli. In experimental fear conditioning studies, fear is thus established by repeatedly pairing a formerly neutral conditioned stimulus (CS; e.g., a geometrical shape) with an aversive US, a procedure known as fear acquisition training (for details, see Ref.^16^). Avoidance of Pavlovian fear CSs (i.e., CS-avoidance) despite costs can thus be studied as a proxy of clinical avoidance behavior^6^. Importantly, costly avoidance has already been demonstrated in face of Pavlovian CSs that are presented in absence of the US^17,18^ as well as stimuli that are categorically related to a Pavlovian fear CS^19^. These studies nicely illustrate that acquired fear can motivate costly avoidance behavior.

However, little is known about the underlying decision process leading up to this behavior. In previous work, we found elevated avoidance in patients with anxiety disorders during approach-avoidance decision conflicts (i.e., when US-avoidance conflicted with gaining rewards)^10^. Interestingly, reward and threat contingencies were easily acquired and patients did not differ from matched controls during US-avoidance in absence of conflicting rewards. This suggests that elevated costly avoidance in patients is at least to some degree linked to deficits in the decision process during approach-avoidance conflicts rather than merely to deficits in learning^10^. Disentangling decision-making from learning is best done by providing subjects with all relevant information on the available options before they take a decision (i.e., learning is not required).

To gain a thorough understanding of the decision process underlying approach-avoidance behavior, it is further essential to examine how this decision unfolds over time. Mouse-tracking (i.e., measuring participants’ computer mouse movements) is a cost-effective and easy to implement method that can provide unique insights into such temporal decision processes (for an overview, see Ref.^20^). Typically, mouse-tracking is implemented within binary choice tasks in which participants choose between two response options by moving the mouse cursor from the bottom center of the screen to one out of two response boxes located in the right and left upper corners^20,21^. The recorded movement trajectories can be used as an index of underlying cognitive processes, for instance, the dynamic variation in which option is favored over time^22^. A variety of analytical techniques can be applied to the acquired real-time data^23,24^. Importantly, a time-continuous multiple regression^25^ (TCMR) allows to uncover how strong the impact of different contributing factors (i.e., predictors) on the mouse movements is at different time points during the decision process. In a previous study, we provided preliminary findings on the temporal impact of threat and reward during the decision process that precedes approach-avoidance decisions^12^. Within a decision-making paradigm, participants repeatedly chose between two response options by moving the mouse cursor to the upper left or right corner of the screen. A threat/high-reward option was always associated with a varying probability of receiving an aversive electrical stimulus (US) but also a high reward of varying magnitude. A safe/low-reward option was associated with the certain absence of the US and a constant low reward. Results showed a fast and strong temporal impact of threat information and a slower and weaker impact of reward information, thereby providing first insights into the dynamic interplay between and relative weighting of reward and threat information during approach-avoidance decision-making. Further research is needed to test if similar temporal dynamics emerge if costly avoidance is directed at a Pavlovian fear CS instead of an innately aversive US. A faster and stronger impact of Pavlovian fear that persists under perceived but actual absence of threat (i.e., the aversive US) and despite missing out on larger rewards may represent a pathway for the development of pathological avoidance.

The current study thus examined approach-avoidance decisions and their temporal dynamics when avoiding a Pavlovian fear stimulus is in conflict with gaining rewards. The study comprised two phases. In the first phase, participants of the aversive learning group were presented with repeated pairings of a formerly neutral visual conditioned stimulus (CS+) and an aversive electrical stimulus (US) to acquire conditioned fear. Another neutral visual stimulus (CS−) was never paired with the US. The neutral learning group underwent a similar procedure but was presented with a neutral tone instead of the aversive US. In the second phase, both groups completed an approach-avoidance paradigm, in which a CS−/low reward option was linked to the certain absence of the CS+ and a small reward. A CS+/high reward option was associated with the potential occurrence of the unreinforced CS+ (i.e., in absence of the US/tone) but also higher rewards. We predicted that information on CS+ probability (Pavlovian fear information) and competing reward magnitude (reward information) would interactively guide approach-avoidance decisions within the aversive learning group. Importantly, we recorded participants’ computer mouse movements to examine the temporal impact of Pavlovian fear and reward information. Based on previous findings^12^, we hypothesized that a stronger and faster impact of fear compared to reward information would be evident in the aversive learning group. We also explored whether decisions of the aversive learning group changed across the paradigm due to extinction learning (i.e., decrement in conditioned responding to the CS+ due to its repeated unreinforced presentation; for details, see Ref.^16^). Finally, we explored whether and how the temporal dynamics underlying these decisions changed across the approach-avoidance paradigm.

## Methods

### Participants

Based on the sample size of a previous study^12^, we aimed at a sample size of 40 participants per group (i.e., 80 in total). Accordingly, 83 healthy participants were recruited from the general community and the students of the University of Würzburg. Exclusion criteria comprised cardiovascular or respiratory diseases, neurological disorders, diagnosed bipolar disorder or depression, psychosis, traumatic brain injury, intellectual disability, substance dependence or abuse, current use of psychotropic drugs, any serious health condition, medical advice to avoid stressful situations, and pregnancy. Advanced psychology students (> four semesters) were also not eligible for participation. In addition, only individuals between 18 and 40 years of age were recruited due to a potential bias of higher age on decision-making^26,27^.

Three participants had to be excluded from data analysis, one for revealing an exclusion criterion after the assessment, one due to technical issues, and one for not following instructions. Thus, the final sample consisted of 80 individuals with 40 participants randomly assigned to the aversive learning group and the neutral learning group. All participants provided written informed consent to the procedures approved by the ethics committee of the Technische Universität Dresden (EK304072015, project B2). All procedures were performed in accordance with the ethical guidelines of the German Psychological Society (DGPs).

Table 1 shows socio-demographic and questionnaire data. Groups did not differ significantly regarding age, sex, state and trait anxiety, depression, stress, impulsiveness, psychological flexibility, and risk-taking. In both groups, there were no outliers in terms of trait anxiety.

**Table 1:** Means (and standard deviations) for socio-demographic and questionnaire data.

		Neutral learning group (n = 40)	Aversive learning group (n = 40)	*t* or *χ*^2^	*p*	*d* or *r*	95% CI for *d* or *r*	
							Lower	Upper
Age		23.90 (4.32)	25.07 (4.89)	1.14^a^	0.258	0.25	− 0.19	0.69
Sex = Female (%)		30 (75.0%)	27 (67.5%)	0.55^b^	0.459	0.08	− 0.12	0.27
Trait anxiety (STAI-Trait)		37.58 (7.67)	35.74 (7.65)	− 1.07^a^	0.288	− 0.24	− 0.68	0.20
State anxiety	STAI-State	33.12 (4.22)	34.20 (6.99)	0.84^a^	0.405	0.19	− 0.25	0.63
	DASS-A	4.95 (4.75)	4.10 (5.26)	− 0.76^a^	0.450	− 0.17	− 0.61	0.27
Depression (DASS-D)		4.90 (4.53)	5.90 (6.02)	0.84^a^	0.404	0.19	− 0.25	0.63
Stress (DASS-S)		7.55 (6.86)	9.25 (8.66)	0.97^a^	0.334	0.22	− 0.22	0.66
Impulsiveness (BIS-15)		71.70 (9.74)	71.68 (9.24)	− 0.01^a^	0.994	0.00	− 0.44	0.44
Psychological flexibility (AAQ-II)		18.68 (7.85)	17.68 (8.44)	− 0.55^a^	0.585	− 0.12	− 0.56	0.32
Risk-taking (R-1)		4.03 (1.31)	4.20 (1.07)	0.65^a^	0.514	0.15	− 0.29	0.58
**Ratings after completion of the approach-avoidance paradigm**								
Aversiveness of electrical stimulation/neutral tone		9.20 (13.93)	71.47 (19.03)	16.70^a^	< 0.001	3.73	3.00	4.46
Positive evaluation of monetary rewards during the paradigm		65.22 (22.03)	59.95 (22.92)	− 1.05^a^	0.297	− 0.23	− 0.67	0.21

*STAI* State-Trait Anxiety Inventory^29^, *DASS* Depression Anxiety Stress Scales^31^, *BIS-15* Barratt Impulsiveness Scale^32^, *AAQ-II* Acceptance and Action Questionnaire II^30^, *R-1* short-scale risk-taking-1^28^.

^a^*t*(78) with Cohen’s *d*.

^b^*χ*^2^(1, 80) with *r.*

### Materials, apparatus, and procedure

After providing written informed consent, participants were free to choose whether they wanted to use their right or left hand to control the computer mouse and the mouse position was adjusted accordingly. All but two participants preferred to use the right hand (39 out of 40 in each of the two groups). Electrodes for skin conductance measurement were attached to the non-mouse hand. Subsequently, participants completed a questionnaire battery assessing socio-demographic data as well as individual differences that might affect approach-avoidance behavior. Specifically, general risk-taking (short-scale risk-taking-1^28^), state and trait anxiety (STAI^29^), psychological flexibility (AAQ-II^30^), anxiety, depression and stress (DASS-21^31^) and impulsiveness (BIS-15^32^) were assessed. Next, a bar-electrode used to deliver the aversive electrical stimulation was attached to the non-mouse forearm of participants in the aversive learning group. The electrical stimulus was generated using a Digitimer DS7A stimulator (Digitimer Ltd) and consisted of 125 consecutive 2-ms stimulations with a total duration of 625 ms. Stimulus intensity was calibrated for each participant in the aversive learning group. To this end, participants were repeatedly asked to rate the aversiveness of the electrical stimulus on a scale from 1 (*not unpleasant*) to 5 (*too unpleasant*). Starting with an intensity of 0.20 mA, the electrical stimulus was stepwise adjusted according to participants’ aversiveness ratings until it reached an intensity that was perceived as clearly unpleasant but bearable and not painful (i.e., an aversiveness rating of 4 out of 5). This resulted in a mean final intensity of 0.73 mA (*SD* = 0.41 mA). As participants in the neutral learning group did not receive any electrical stimuli, no calibration was carried out. Next, the aversive learning group underwent an aversive fear acquisition training. The neutral learning group completed a similar training phase, however, with a neutral outcome instead of an aversive stimulation.

#### Acquisition training

Participants in the aversive learning group completed three blocks of differential fear acquisition training. In each block, each of two CSs was presented four times (i.e., 12 trials per CS in total). An orange triangle (CS+) was paired with the aversive electrical stimulus (unconditioned stimulus, US) in 75% of the acquisition trials. A purple hexagon (CS−) was never paired with the US. The neutral learning group underwent the same procedure, except that they received a neutral 440 Hz tone presented at a comfortable volume with a duration of 625 ms instead of the aversive electrical stimulus.

At the beginning of each trial, a small-scale version of the corresponding CS (150 × 150 pixels) was presented until participants indicated how likely they expect to receive an US/a tone during the full-scale presentation of the respective CS on a visual analog scale of 0 to 100% (expectancy rating). Subsequently, the full-scale CS (300 × 300 pixels) was presented for 7 s. In case of a reinforced CS+ trial, the US/tone was delivered at a random point in time between 6800 and 6900 ms after CS onset. The next trial started following an inter-trial interval between 15 and 18 s. Expectancy ratings prior to full-scale CS presentation served as indicators of contingency learning. Skin conductance responses (SCRs) during presentation of CS+ and CS− were assessed as physiological indicators of fear learning.

#### Approach-avoidance paradigm

Following acquisition training, participants completed an approach-avoidance decision paradigm that was identical for both groups. The paradigm was based on a previous study^12^. In each of 324 trials, participants chose between two different options: A CS−/low reward option was always linked to the certain absence of the CS+ (0%) and a small, fixed reward (25 Cents). A CS+/high reward option was associated with varying probabilities of a presentation of the CS+ (0%, 10%, 25%, 45%, 70% or 100%) and varying, higher rewards (28, 31, 36, 42, 50, 62, 83, 125 or 250 Cents). All possible combinations of reward magnitude and CS+ probability (54 combinations) were presented six times in a randomized order. Participants were informed that the presented rewards were hypothetical in nature. Previous studies have verified that hypothetical rewards entail a sufficient appetitive value to modify avoidance behavior in face of feared or aversive stimuli^12,17,33,34^. Frequency of choosing the CS−/low reward option was used as a measure of costly CS+ avoidance.

**Figure 1:**
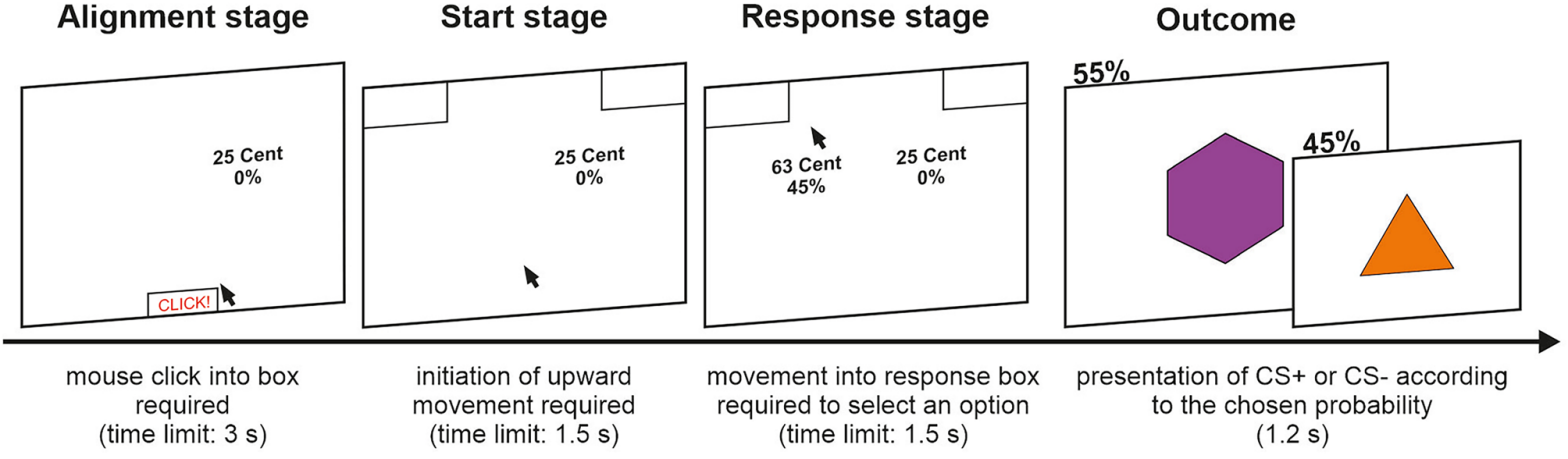
Schematic representation of the trial sequence. First, the CS−/low reward option was presented, and participants had to click into a small box at the bottom of the screen (time limit: 3 s). Second, an upward movement was required until the mouse cursor crossed an invisible line 50 pixels above the starting position (time limit: 1.5 s). Third, the CS+/high reward option appeared, and participants could choose one of the options by moving the mouse cursor to the response box of their preferred option (time limit: 1.5 s). Finally, the CS+ (orange triangle) was presented with the chosen probability (duration: 1.2 s). In trials without CS+ presentation, the CS− (purple hexagon) was shown.

Moreover, we recorded participants’ computer mouse movements to examine the temporal dynamics of the decision process underlying approach-avoidance (i.e., the temporal impact of CS+ probability and reward magnitude). To this end, each trial followed a standardized sequence (see Fig. 1): At the beginning, the CS−/low reward option appeared counterbalanced on either the right or the left side of the screen, giving participants the opportunity to process this option in advance. Participants had to click into a small box at the bottom middle of the screen within a time limit of 3 s to ensure a fixed starting position (Alignment stage). Then, participants had to perform an upward mouse movement within a time limit of 1.5 s until the mouse cursor crossed an invisible horizontal line 50 pixels above the starting position (Start stage). This stage was not restricted to vertical movements, i.e., participants were able to freely move the mouse cursor. This ensured that a naturalistic mouse movement was initiated before the next phase commenced. As soon as they passed this boundary, the CS+/high reward option appeared on the opposite side of the screen and participants had to choose one of the options by moving the mouse cursor to one of the response boxes in the left or right corner of the screen within a time limit of 1.5 s (Response stage). The respective time limits were implemented to ensure continuous mouse movements (i.e., there was no time to pause mouse movement during the decision process). In addition, participants were instructed to perform smooth, upward mouse movements without stopping until they reached one of the response boxes. Before starting the approach-avoidance paradigm, the experimenter demonstrated the correct execution of the mouse cursor movements and participants completed 40 practice trials without CS presentation. If participants exceeded a time limit, an error message was shown for 0.75 s following the related stage and the respective trial was cancelled. Such error trials were immediately re-randomized to obtain data for all 324 trials.

According to the chosen option, the CS+ was presented for 1.2 s with the indicated probability followed by an inter-trial interval of 0.5 s. In trials without CS+ presentation, the CS− was shown for the same duration. No USs or tones were presented during the decision task, i.e., all CSs were unreinforced. Thus, the paradigm measured CS-avoidance (instead of US-avoidance; see Ref.^6^). Importantly, participants did not receive any instructions concerning CS-US contingencies. Therefore, they did not know beforehand that no USs or tones would be presented throughout the task.

### Data acquisition and preprocessing

#### Skin conductance responses

Skin conductance was measured during acquisition training via two reusable Ag/AgCl electrodes attached to the hypothenar eminence of the participants’ non-mouse hand and a V-Amp 16 amplifier (Brain Products GmbH, Gilching, Germany). The sampling rate was set to 1000 Hz. Data preprocessing was conducted using BrainVision Analyzer 2 software (Brain Products GmbH, Gilching, Germany). Raw data were filtered with a 50 Hz notch filter and a 1 Hz low-pass filter. SCR amplitudes were obtained by semi-manual trough-to-peak scoring. Negative SCRs were set to zero and included in the analyses. Amplitudes were then square root transformed to reduce skewness. Two participants of the neutral learning group had to be excluded from SCR analysis due to poor signal quality.

#### Mouse movement trajectories

Computer mouse movement trajectories (i.e., time series of x-coordinates and y-coordinates) were recorded during the Response stage of the approach-avoidance paradigm with a sampling rate of 100 Hz. During preprocessing, trajectories ending on the right-hand side were flipped to the left so that all movements end in the same direction. Next, movement trajectories were realigned to a common starting position. Moreover, each trajectory was time-normalized to 100 time slices of equal length.

The area between a recorded trajectory and an ideal straight line from start to end (area under the curve; AUC) was calculated as a measure of response conflict^20^ using R^35^ and the *mousetrap* package^24^. For temporal dynamics analyses, the angle of movement relative to the y-axis was calculated for each time slice using Matlab 2015b (The MathWorks Inc., Natick, Massachusetts, United States). Each movement angle corresponds to the instantaneous direction of the mouse cursor during a specific time slice (i.e., the momentary movement tendency towards or away from a given option; for details, see Ref.^25^). Mouse movement trajectories of all participants were entered into statistical analysis. In previous research, individuals that favored one of the response options in the vast majority of trials were excluded from mouse-tracking analyses (e.g., Refs.^12,36^). Importantly, supplementary analyses of temporal dynamics revealed that our results did not qualitatively change when following this approach by excluding participants with a proportion of CS+/high reward choices > 90% or < 10% (see Supplementary Figs. S1, S2 and Supplementary Tables S1, S2).

### Statistical analysis

Statistical analysis was conducted using JASP (Version 0.8.5.1^37^), R^35^ and Matlab 2015b (The MathWorks Inc., Natick, Massachusetts, United States). For analyses of variance (ANOVAs), Greenhouse–Geisser correction was applied in case of violated sphericity.

#### Contingency learning and fear acquisition

Manipulation check analyses aimed to verify contingency learning in both groups, but fear acquisition in the aversive learning group only. For contingency learning, expectancy ratings were analyzed using an ANOVA with the between subject factor Group and the within-subject factors CS type and Block. For fear acquisition, SCR amplitudes were entered into an ANOVA with the between-subject factor Group and the within-subject factors CS type and Block.

#### Approach-avoidance decisions

Participants’ decisions within the approach-avoidance paradigm represent a binary outcome (i.e., either a CS−/low reward or a CS+/high reward choice), thus generalized linear mixed models (GLMMs) were particularly suitable for analysis. Accordingly, GLMMs were calculated using R^35^ as well as the packages *lme4* and *afex*. GLMMs were fit by maximum likelihood (Laplace Approximation) with binomial error distribution and the logit link function, which accounts for the binary nature of the data. Continuous predictors were centered (*M* = 0) and scaled (*SD* = 1) prior to analysis and correlations among random terms were disabled. Likelihood ratio tests were applied to obtain p-values for all fixed effects. Follow-up analyses for GLMMs were calculated using the R package *emmeans*.

First, decisions of all participants were analyzed using a GLMM. Fixed effects comprised the continuous predictors *Reward Magnitude* of the CS+/high reward option and *CS*+ *Probability,* the categorical predictor *Group* as well as all two-way interactions. In addition, a by participant random intercept as well as by participant random slopes for *Reward Magnitude* and *CS*+ *Probability* were included. A more complex model including the three-way interaction of all predictors yielded the same significant main effects and two-way interactions, however the three-way interaction was non-significant and thus not included in the final model.

Next, to explore whether decisions of the aversive learning group changed across the approach-avoidance paradigm due to fear extinction processes, we ran the same GLMM with the additional continuous predictor *Trial Repetition* for the aversive learning group only. This predictor counted the number of times a specific trial (i.e., a combination of reward magnitude and CS+ probability) had been presented during the task (1st to 6th repetition). As before, fixed effects included all possible two-way interactions. Further, a by participant random slope for *Trial Repetition* was introduced. A more complex model including the three-way interaction of all predictors did not converge successfully but yielded the same significant main effects and two-way interactions.

#### Mouse movement trajectories

We explored within the aversive learning group whether conflict strength differed when choosing either the CS+/high reward or the CS−/low reward option. Accordingly, mean AUC values of the aversive learning group were entered into an exploratory within subject *t*-test comparing CS+/high reward and CS−/low reward choices.

To examine the temporal impact of reward and Pavlovian fear information during the decision process, mouse movement angles of the aversive learning group were analyzed using a time-continuous multiple regression (TCMR; for details, see Ref.^25^). In addition, a second TCMR was applied to analyze mouse movements of the neutral learning group. The TCMRs were conducted using the freely available TCMR toolbox for Matlab^25,38^: *Reward Magnitude* of the CS+/high reward option and *CS*+ *Probability* were used as predictors. Both predictors were normalized to an interval of − 1 to 1 to obtain comparable beta weights. Next, a multiple regression with these two predictors and movement angle as dependent variable was calculated for each time step within each participant, resulting in two time-varying beta weights for each participant (2 predictors × 100 time steps). *T*-tests were applied to test the beta weights for each predictor at each time step against zero. Only segments of more than 10 consecutive significant beta weights were considered as meaningful to correct for multiple comparisons. This criterion was adopted to stay consistent with previous research^12^ (for Monte Carlo analyses on this issue, see Refs.^39,40^). In sum, this analysis reveals how strong the impact of each predictor is at different time points during the decision process.

Moreover, we performed an exploratory analysis to gain additional insights into the temporal dynamics of costly CS-avoidance in the aversive learning group. Specifically, we conducted a separate TCMR for each half of the approach-avoidance paradigm to test for changes in the temporal impact of Pavlovian fear and reward information due to extinction processes (1^st^ half = 1^st^ to 3^rd^ repetition of each trial; 2^nd^ half = 4^th^ to 6^th^ repetition). The resulting beta weights for each time step and each predictor were then entered into within subject *t*-tests to explore potential changes in the temporal dynamics between the first and the second half of the paradigm. As above, only segments of more than 10 successive significant *t*-tests were accepted as meaningful.

## Results

### Acquisition training

#### Expectancy ratings as indicators of contingency learning

In both groups, expectancy ratings during acquisition training were higher for CS+ than for CS−. Furthermore, expectancy ratings to CS+ increased across acquisition whereas expectancy ratings to CS− decreased. This effect was indicated by a significant interaction of CS type and Block, *F*(1.76,137.53) = 86.95, *p* < 0.001, η_p_^2^ = 0.527. However, there were no significant main or interaction effects involving Group, *F*s ≤ 1.35, *p*s ≥ 0.248.

In sum, both groups learned across blocks that the CS+ but not the CS− was linked to the potential presentation of the electrical stimulus or the neutral tone. Thus, both groups showed successful contingency learning.

#### SCRs as physiological indicators of fear learning

During acquisition training, SCRs were stronger during CS+ presentations than during CS− presentations in the aversive learning group, but not in the neutral learning group. This effect was indicated by a significant interaction of CS type and Group, *F*(1,76) = 39.67, *p* < 0.001, η_p_^2^ = 0.343. Importantly, Bonferroni corrected follow-up *t*-tests revealed in the aversive learning group significantly higher SCRs to CS+ (*M* = 0.58, *SD* = 0.32) than to CS− (*M* = 0.43, *SD* = 0.29), *t*(39) = 6.11, *p* < 0.001, *d* = 0.97, CI_95_ = 0.59–1.34. In the neutral learning group, no significant difference between CS+ (*M* = 0.34, *SD* = 0.26) and CS− (*M* = 0.38, *SD* = 0.28) was observed, *t*(37) = − 2.33, *p* = 0.051, *d* = − 0.38, CI_95_ = − 0.71 to − 0.05. Moreover, SCRs decreased across acquisition as indicated by a significant main effect of Block, *F*(2,152) = 14.90, *p* < 0.001, η_p_^2^ = 0.164.

Overall, SCRs were higher to CS+ than to CS− in the aversive learning group, which provides evidence for successful fear acquisition. As expected, this differentiation was not observed in the neutral learning group.

### Approach-avoidance paradigm

#### Costly avoidance of the Pavlovian fear stimulus

**Figure 2:**
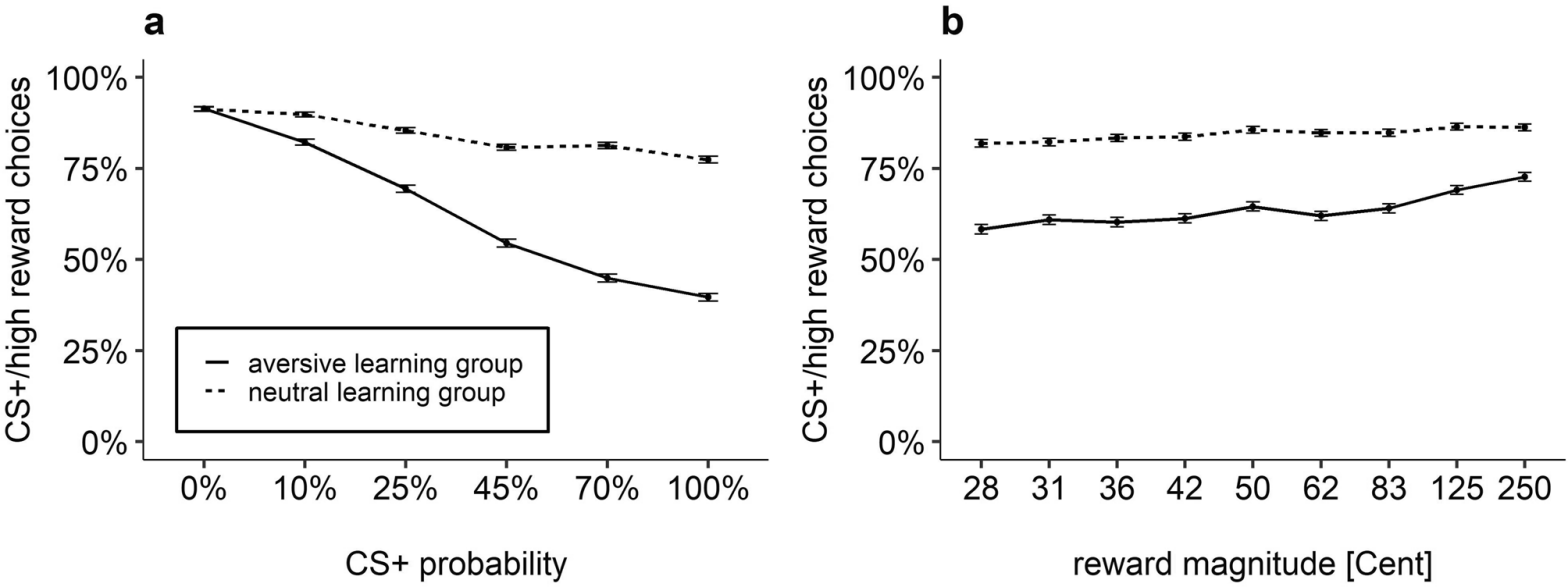
Mean percentage of CS+/high reward choices (with standard error) in relation to (**a**) CS+ probability and (**b**) reward magnitude of the CS+/high reward option for the neutral and the aversive learning group.

Participants of the aversive learning group chose the CS+/high reward option in 63.6% of the trials (*SD* = 24.5%); participants of the neutral learning group chose this option in 84.3% of the trials (*SD* = 20.9%). In the aversive learning group, frequency of CS+/high reward choices systematically decreased with increasing probability of a CS+ presentation (see Fig. 2). In contrast, the neutral learning group rarely selected the CS−/low reward option irrespective of CS+ probability. This effect was indicated by a significant interaction of the predictors *Group* and *CS*+ *Probability*, *χ*^2^(1) = 14.60, *p* < 0.001. A stronger effect of CS+ probabilities in the aversive learning group was found in a follow-up analysis: The predicted trend for *CS*+ *Probability* was more negative in the aversive learning group (= − 1.83; CI_95_ = − 2.37 to − 1.29) compared to the neutral learning group (= − 0.24; CI_95_ = − 0.73 to 0.31). Bonferroni corrected follow-up comparisons of estimated marginal means (EMMs) yielded no significant difference between groups for the three lowest CS+ probabilities (0%, 10%, 25%), |*z|*s ≤ 2.28, *p*s ≥ 0.136. Importantly, however, participants in the aversive compared to the neutral learning group more frequently avoided the CS+/high reward option when it was linked to higher CS+ probabilities (45%, 70%, 100%), |*z|*s ≥ 3.52, *p*s ≤ 0.003.

**Figure 3:**
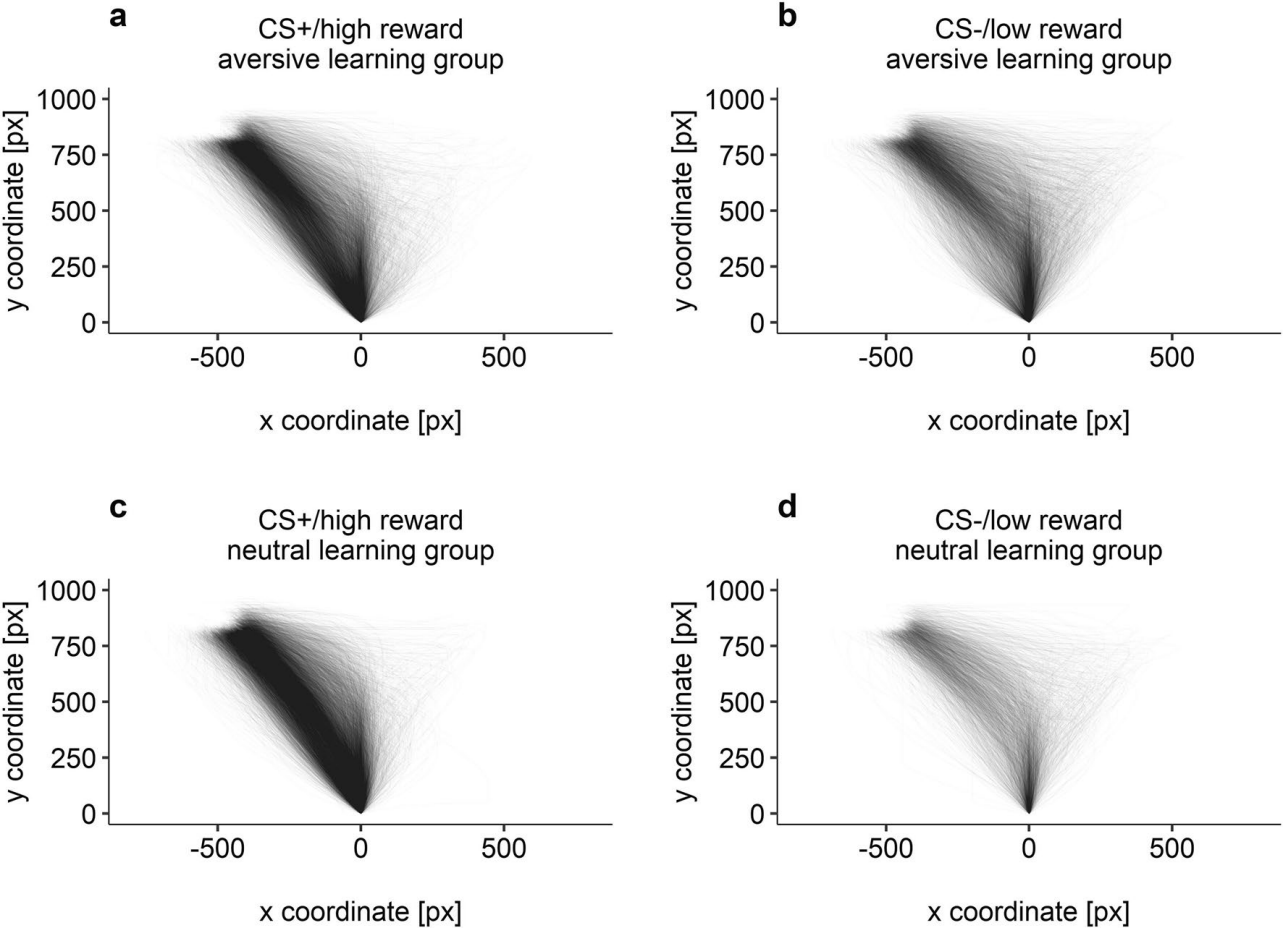
Individual movement trajectories for CS+/high reward choices in the aversive learning group (**a**), CS−/low reward choices in the aversive learning group (**b**), CS+/high reward choices in the neutral learning group (**c**), and CS−/low reward choices in the neutral learning group (**d**). All trajectories were flipped to the left, aligned to a common starting position and time-normalized.

Overall, the frequency of CS+/high reward choices increased with increasing reward magnitudes (see Fig. 2). Accordingly, we found a significant effect of the predictor *Reward Magnitude*, *χ*^2^(1) = 26.22, *p* < 0.001 with a slope of 0.39 (CI_95_ = 0.25–0.53). It seems that this effect was mainly driven by the aversive learning group (see Fig. 2). However, the two-way interaction of the predictors *Reward Magnitude* and *Group* was non-significant, *χ*^2^(1) = 0.50, *p* = 0.481. There was also no significant interaction of the predictors *Reward Magnitude* and *CS*+ *Probability, χ*^2^(1) = 3.51, *p* = 0.061*.*

In sum, participants in the aversive learning group showed increased CS-avoidance with increasing CS+ probability. Thus, fear acquisition resulted in significant costly CS+ avoidance. However, avoidance decreased as rewards for approach increased.

As shown by the previous analyses, the frequency of CS+/high reward choices in the aversive learning group decreased with increasing probability of a CS+ presentation. However, this decrease became smaller across trial repetitions (see Supplementary Fig. S3). This effect was indicated by a significant interaction of the predictors *CS*+ *Probability* and *Trial Repetition*, *χ*^2^(1) = 4.78, *p* = 0.029. A follow-up analysis yielded a more negative slope for *CS*+ *Probability* at the beginning of the task (slope for the 1st repetition of each trial = − 2.02), which consistently decreased across trial repetitions (2nd repetition: − 1.97, 3rd repetition: − 1.92, 4th repetition: − 1.88, 5th repetition: − 1.83, last repetition: − 1.78). In line with this finding, EMMs describe an increasing frequency of CS+/high reward choices across the task in the aversive learning group (predicted probabilities for CS+/high reward choices: 1st trial repetition: 54.3%, 2nd repetition: 57.7%, 3rd repetition: 61.2%, 4th repetition: 64.6%, 5th repetition: 68.0%, 6th repetition: 71.4%). In other words, avoidance increased less strongly along higher CS+ probability across the task.

Across trial repetitions, final decisions of the aversive learning group were further more strongly affected by the varying reward magnitudes. This effect was indicated by a significant interaction of the predictors *Reward Magnitude* and *Trial Repetition*, *χ*^2^(1) = 5.04, *p* = 0.025. A follow-up analysis yielded a positive slope for *Reward Magnitude* at the beginning of the task (slope for the 1st repetition of each trial = 0.38), which subsequently increased across trial repetitions (2nd repetition: 0.42, 3rd repetition: 0.46, 4th repetition: 0.50, 5th repetition: 0.54, last repetition: 0.59).

Accounting for trial repetitions in the aversive learning group further revealed that the impact of the varying CS+ probabilities on the proportion of CS+/high reward choices was attenuated in the presence of higher competing rewards. This effect was indicated by a significant interaction of the predictors *CS*+ *Probability* and *Reward Magnitude*, *χ*^2^(1) = 5.50, *p* = 0.019. A follow-up analysis yielded a negative slope for *CS*+ *Probability* for each individual reward magnitude. But importantly, this slope became less steep as reward magnitudes increased (28 Cent: − 1.96; 31 Cent: − 1.96; 36 Cent: − 1.95; 42 Cent: − 1.95; 50 Cent: − 1.94; 62 Cent: − 1.92; 83 Cent: − 1.89; 125 Cent: − 1.84; 250 Cent: − 1.69).

In sum, in the aversive learning group, CS-avoidance decreased across the approach-avoidance paradigm, which was linked to a decreasing impact of CS+ probability on participants’ final decisions, but also an increasingly strong impact of reward magnitudes. This change in decision-making suggests that extinction learning towards the CS+ took place.

#### Area under the curve within the aversive learning group

In the aversive learning group, AUC values were larger for CS−/low reward choices (*M* = 85,193.30, *SD* = 51,117.14) compared to CS+/high reward choices (*M* = 57,768.52, *SD* = 41,867.08), *t*(37) = 2.94, *p* = 0.006, *d* = 0.48, CI_95_ = 0.14–0.81. Thus, movement trajectories deviated more from an ideal straight line from start to end towards the non-chosen option if participants chose the CS−/low reward option (see Fig. 3). Two participants of the aversive learning group had to be excluded from this analysis, one for choosing the CS−/low reward option in all trials and the other for choosing the CS+/high reward option in all trials (i.e., they did not provide movements for both options). Participants of the neutral learning group were not included in this analysis, since as expected, only few movements towards the CS−/low reward option were performed (see Figs. 2 and 3).

#### Temporal dynamics

**Figure 4:**
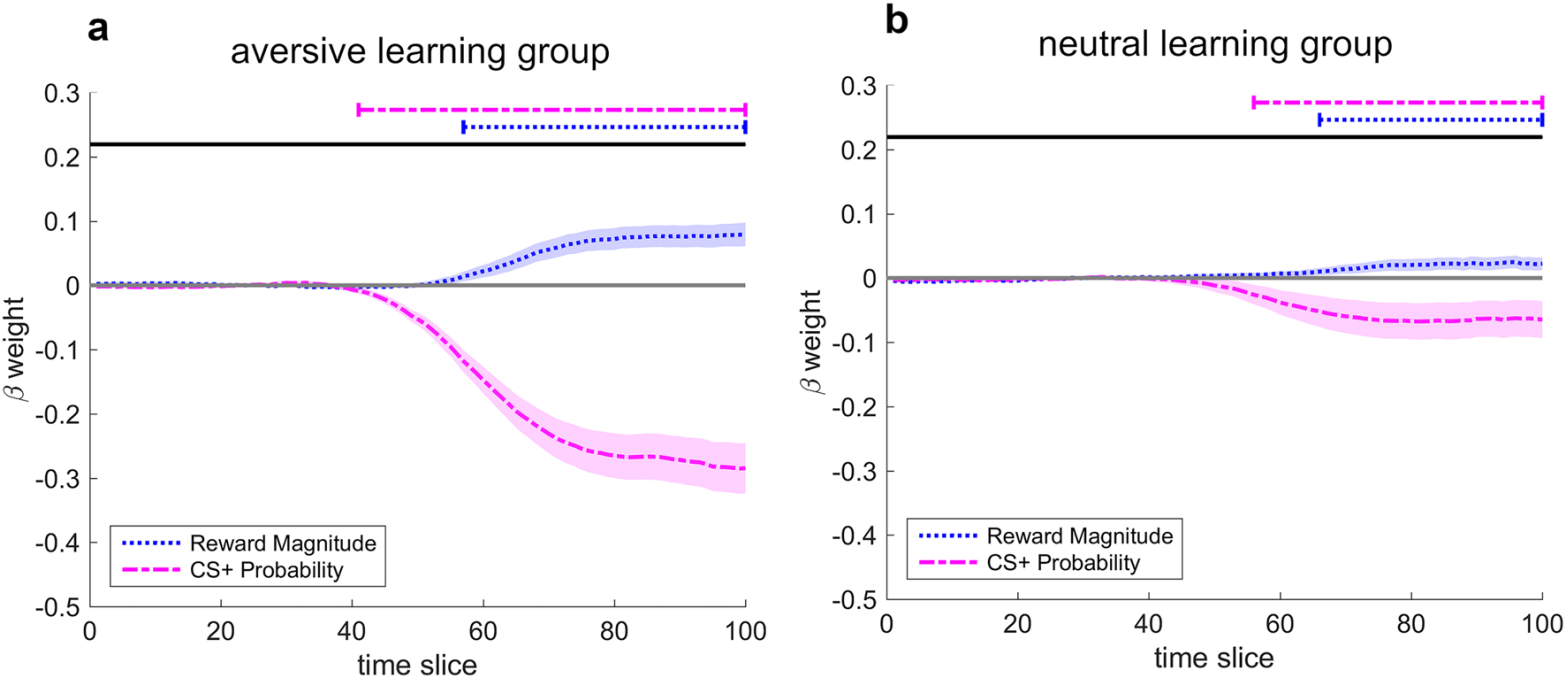
Time-continuous beta weights from TCMR analysis in (**a**) the aversive learning group and (**b**) the neutral learning group for the predictors Reward Magnitude and CS+ Probability. Positive β weights indicate movement tendencies towards the CS+/high reward option, negative β weights indicate tendencies towards the CS−/low reward option. Horizontal lines at the top indicate segments of significant impact. Shaded areas represent standard error of the mean. Only segments of more than 10 successive significant *t*-tests were accepted as meaningful. In the aversive learning group, a time slice corresponds on average to 7.8 ms. In the neutral learning group, a time slice corresponds on average to 7.7 ms.

Results of the TCMR analysis for the aversive learning group are displayed in Fig. 4 and Table 2. During the decision process, both Reward Magnitude and CS+ Probability showed a significant impact: Higher probabilities of a presentation of the CS+ predicted movement tendencies towards the CS−/low reward option; higher competing rewards predicted tendencies towards the CS+/high reward option. The impact of CS+ Probability was considerably stronger and emerged earlier compared to the impact of Reward Magnitude (see Fig. 4 and Table 2).

Results of the TCMR analysis for the neutral learning group are displayed in Fig. 4 and Table 2. As in the aversive learning group, both Reward Magnitude and CS+ Probability showed a significant impact. The impact of CS+ Probability emerged again earlier than the impact of Reward Magnitude. But importantly, the beta weights of both predictors remained rather small throughout the decision process (see Fig. 4 and Table 2).

In sum, the TCMR analysis for the aversive learning group provides evidence for a stronger weighting of Pavlovian fear information towards avoidance compared to competing reward information, which was weighted towards approach. In the neutral learning group, in contrast, the impact of the neutral CS as well as reward information remained small throughout the decision process.

**Figure 5:**
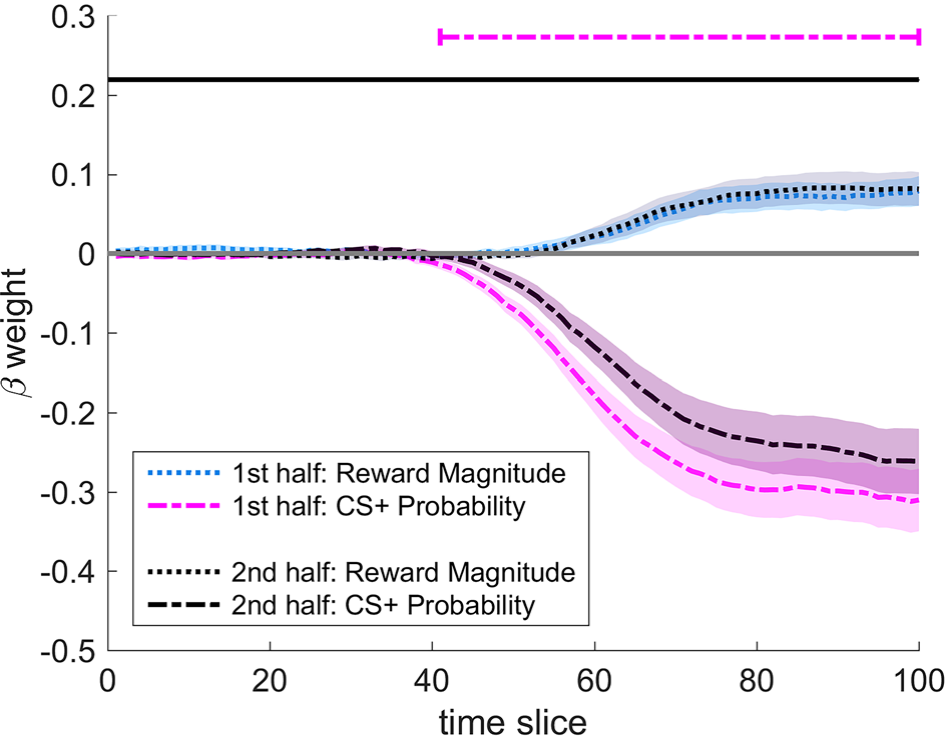
Time-continuous beta weights from TCMR analysis for the predictors Reward Magnitude and CS+ Probability for the first half (light-colored) and the second half (dark-colored) of the approach-avoidance paradigm in the aversive learning group. Positive β weights indicate movement tendencies towards the CS+/high reward option, negative β weights indicate tendencies towards the CS−/low reward option. Horizontal lines at the top indicate segments of significant difference between both halves for a specific predictor. Shaded areas represent standard error of the mean. Only segments of more than 10 successive significant *t*-tests were accepted as meaningful. In the aversive learning group, a time slice corresponds on average to 7.8 ms.

Results of the TCMR analyses for the first and the second half of the approach-avoidance paradigm in the aversive learning group are shown in Fig. 5 and Table 3. In both halves, the impact of CS+ Probability was stronger and emerged earlier compared to the impact of Reward Magnitude. The impact of CS+ Probability, however, was significantly stronger in the first compared to the second half of the paradigm (see Fig. 5). For Reward Magnitude, the comparison between both halves did not yield any segments of significant beta weights.

**Table 2:** Segments of significant beta weights for Reward Magnitude and CS+ Probability in the aversive learning group and the neutral learning group.

Beta for	Aversive learning group				Neutral learning group			
	Start	End	Duration	Peak strength	Start	End	Duration	Peak strength
Reward magnitude	57	100	43 (≈ 335.4 ms)	0.079	66	100	34 (≈ 261.8 ms)	0.025
CS+ probability	41	100	59 (≈ 460.2 ms)	0.285	56	100	44 (≈ 338.8 ms)	0.068

In the aversive learning group, a time slice corresponds on average to 7.8 ms. In the neutral learning group, a time slice corresponds on average to 7.7 ms. Only segments of more than 10 significant *t*-tests were accepted as meaningful.

Taken together, the impact of Pavlovian fear information decreased from the first to the second half of the paradigm in the aversive learning group, which can be seen as a behavioral indicator of extinction. But importantly, even in the second half, there was still a strong impact of fear information. The impact of competing reward information, in contrast, did not differ significantly across the two halves. This suggests that the subjective value of the competing rewards remained rather constant across the paradigm.

## Discussion

This study examined approach-avoidance decisions when avoiding a Pavlovian fear stimulus is in conflict with gaining rewards (i.e., costly CS-avoidance). To gain insights into the decision process leading to approaching rewards vs. avoiding the fear stimulus, Pavlovian fear and reward information were explicitly provided during continuous recording of mouse movements required for choosing between options. Main findings demonstrate (a) pronounced costly avoidance of a Pavlovian fear CS, compared to a neutral CS, which was attenuated when rewards for approach were high. (b) Costly avoidance of the fear CS decreased across the task, yet surprisingly strong avoidance behavior persisted until the end of the task even though no USs occurred across 324 trials. (c) Temporal dynamics analyses revealed a noticeably stronger and faster impact of Pavlovian fear information compared to reward information during decision-making. (d) The impact of fear information during decision-making decreased modestly from the first to the second half of the task while the impact of competing reward information remained rather constant. Combined, our results point towards a stronger weighting of Pavlovian fear compared to reward information during approach-avoidance decisions that persists even in absence of objective danger. These findings elucidate how Pavlovian fear guides approach-avoidance decision processes, which can contribute to maladaptive avoidance behavior.

As expected, both the probability of a Pavlovian fear CS as well as the magnitude of competing rewards had a systematic effect on approach-avoidance decisions. Specifically, avoidance increased strongly with increasing probability of a Pavlovian fear CS. Extending previous findings^17,18^, our results demonstrate that such costly CS-avoidance can be surprisingly persistent, despite the complete absence of aversive USs across a high number of trials. This indicates that not only aversive USs but also Pavlovian fear CSs can strongly guide approach-avoidance decisions. Importantly, avoidance of the Pavlovian fear CS decreased when rewards for approach increased, paralleling past findings on US-avoidance^11–13^. This underlines the importance of avoidance costs and the potential usefulness of rewards, which can help to counter maladaptive clinical avoidance (see also Ref.^6^). In contrast to the fear CS, the neutral CS (i.e., the CS that was paired with a neutral tone) was more often approached, irrespective of CS probability. Notably, both groups perceived the monetary rewards as positive, without significant group differences. More frequent approach towards the neutral CS can therefore not be explained by a more positive reward evaluation in the neutral learning group. Thus, acquired fear motivated costly avoidance.

**Table 3:** Segments of significant beta weights for Reward Magnitude and CS+ Probability separately for the first and the second half of the approach-avoidance paradigm in the aversive learning group.

Beta for	First half				Second half				First vs. second half		
	Start	End	Duration	Peak strength	Start	End	Duration	Peak strength	Start	End	Duration
Reward magnitude	59	100	41 (≈ 319.8 ms)	0.079	57	100	43 (≈ 335.4 ms)	0.083	–	–	–
CS+ probability	40	100	60 (≈ 468.0 ms)	0.312	46	100	54 (≈ 421.2 ms)	0.262	41	100	59 (≈ 460.2 ms)

In the aversive learning group, a time slice corresponds on average to 7.8 ms. Only segments of more than 10 significant *t*-tests were accepted as meaningful.

An exploratory analysis further revealed that although costly avoidance of the Pavlovian fear CS decreased over trials, significant avoidance persisted. Specifically, CS-avoidance decreased across trial repetitions while decisions were increasingly affected by competing rewards. Yet, significant avoidance behavior persisted even towards the end of the task. This is surprising, since participants never experienced an aversive US in a large number of trials (N = 324). One potential explanation could have been that participants rarely encountered the Pavlovian fear CS as a result of strong avoidance and thus never experienced the absence of the US after the fear CS. However, most participants experienced the unreinforced fear CS several times, and thus, had the opportunity for fear extinction learning (see Supplementary Fig. S4). An alternative explanation might thus be that little extinction learning occurred despite the unreinforced fear CSs. Specifically, threat expectancies towards the fear CS might have remained high despite its unreinforced presentation. Previous research, however, showed that conditioned fear is typically reduced by a substantially lower amount of extinction trials (e.g., Ref.^41^). Finally, a third possibility is that the Pavlovian fear CS may have acquired a negative valence during fear acquisition training that persisted even when the aversive US no longer occurred (e.g., Ref.^42^). Participants may have continued to avoid since they perceived the Pavlovian fear CS itself as unpleasant. As a limitation of the present paradigm, we did not directly measure threat expectancy or negative valence. It therefore does not allow to disentangle whether persistent CS-avoidance was mainly driven by negative valence of the Pavlovian fear CS or persistent threat expectancy. Further research on the driving factors of costly CS-avoidance is warranted.

Temporal dynamics analyses shed further light on how Pavlovian fear is integrated in the underlying approach-avoidance decision process. In particular, a considerably stronger and faster impact of the Pavlovian fear CS compared to competing reward information was found during decision-making. The difference in strength between Pavlovian fear and reward information was pronounced (i.e., the peak beta weight of CS+ Probability was ~ 0.29, while Reward Magnitude reached a maximum of ~ 0.08). Thus, information on fear CS probability was the major determinant during decision-making, as indicated by participants’ computer mouse movements. In the neutral learning group, the impact of the neutral CS as well as reward information remained small throughout the decision process. This indicates a limited impact of the varying reward and threat information and instead a large proportion of pre-planned choices (e.g., approach irrespective of the presented CS and reward information). These findings were expected as no conflict was established in the neutral learning group. In sum, temporal dynamics analyses highlight that Pavlovian fear guides approach-avoidance decision processes and not only final behavior.

It cannot be ruled out that the stronger impact of Pavlovian fear compared to reward information found in the aversive learning group was partly driven by the selected levels of fear and reward information during the paradigm, which were identical for all participants. Higher rewards, for example, may result in a stronger impact of reward information. Nevertheless, the strong impact of Pavlovian fear information is remarkable as the fear CS was never followed by aversive consequences during the decision task. In fact, this pattern of results closely resembles recent findings on costly US-avoidance. Specifically, a stronger and faster impact of US probabilities compared to competing reward information was found when avoiding an aversive US conflicted with gaining rewards^12^. From an evolutionary perspective, preferential and fast processing of threat and fear-related information during decision-making may represent a cognitive tendency that originally increased an individual’s chance of survival. However, such a tendency may also facilitate the development of pathological avoidance when objective threat is no longer present.

An exploratory temporal dynamics analysis further revealed a reduced impact of Pavlovian fear information in the second half of the task. Interestingly, the impact of reward information remained rather constant. This indicates that the motivational value of the Pavlovian fear CS was specifically reduced, which further supports the idea of some degree of extinction learning across trials. Thus, our results provide preliminary evidence that extinction learning may impact the weighting of Pavlovian fear information during approach-avoidance decisions. Importantly, it also highlights that the weighting of fear and reward information during approach-avoidance decisions is not simply a linear trade-off (i.e., the less Pavlovian fear information is weighted, the more rewards are weighted). Instead, temporal dynamics can deliver unique insights how both types of information independently guide decisions. For example, when the impact of Pavlovian fear decreases but the impact of rewards remains constant, the relative importance of rewards becomes larger. This change in relative importance may help to explain the increasing impact of rewards evident in participants’ final decisions across trial repetitions (as described above). Surprisingly, the impact of Pavlovian fear information decreased only modestly over time (i.e., its peak beta weight was ~ 0.31 in the first half, and ~ 0.26 in the second half). Even in the second half of the task, Pavlovian fear information remained the major determinant of decision-making.

An exploratory analysis of movement trajectories pointed towards a stronger decision conflict when choosing to avoid the fear CS compared to choices in favor of approach. This could imply that avoidance was preceded by decision processes that are characterized by stronger conflict. In our paradigm, approach was more frequent and may thus have been the initial choice tendency. Avoidance due to processing of threat information may then result in higher conflict. Due to the very high levels of approach in the neutral learning group, it remains unclear whether this pattern is specific to fear driven avoidance, which represents a limitation of the current study.

In the present study, costly avoidance of a Pavlovian fear CS was examined using a newly adapted approach-avoidance paradigm. The monetary rewards presented during the paradigm were hypothetical in nature, which might present a limitation for generalizing our findings to tangible rewards. Hypothetical rewards were used since there is evidence that they are well suited to modify avoidance behavior in face of feared or aversive stimuli^12,17,33,34^. In support, a systematic effect of hypothetical rewards on avoidant decision-making was found in the present study. However, it remains to be tested whether the temporal dynamics of costly CS-avoidance change when approach is linked to rewards with a higher motivational value (e.g., real monetary rewards). Thereby it is of particular interest, whether the fast and strong temporal impact of Pavlovian fear information, which was found in the present study, can be replicated in face of highly attractive rewards.

In sum, the present study provides new insights into costly avoidance of Pavlovian fear stimuli and its temporal dynamics. Findings highlight that newly acquired fear can trigger pronounced and persistent costly CS-avoidance, despite the absence of objective danger. Most importantly, a stronger weighting of Pavlovian fear information compared to competing rewards was demonstrated during approach-avoidance decisions, which can contribute to maladaptive clinical avoidance.

## Supplementary Information

Supplementary Information.

## Data Availability

The data generated and analyzed in the current study will be uploaded to the repository of the Open Science Framework (osf.io/tbxug).
