# Supplementary material for: Costly avoidance of Pavlovian fear stimuli and the temporal dynamics of its decision process
Source: Sci Rep. 2022 Apr 21;12:6576. doi: 10.1038/s41598-022-09931-1 (PMC9023480; doi:10.1038/s41598-022-09931-1)
Supplement: Supplementary file 1 — Supplementary Information. [file 41598_2022_9931_MOESM1_ESM.pdf]

Supplementary information for:

Costly avoidance of Pavlovian fear stimuli and the temporal dynamics of its decision process

Juliane M. Boschet[1] ORCID: 0000-0002-2607-912X

Stefan Scherbaum[2] ORCID: 0000-0002-4408-6016

Andre Pittig[1,3,*] ORCID: 0000-0003-3787-9576

[1] Department of Psychology (Biological Psychology, Clinical Psychology, and Psychotherapy), University of Würzburg, Würzburg, Germany.

[2] Department of Psychology, Technische Universität Dresden, Germany.

[3] Translational Psychotherapy, Department of Psychology, Friedrich-Alexander University Erlangen-Nürnberg (FAU), Erlangen, Germany.

Correspondence concerning this article should be addressed to Andre Pittig, Department of Psychology, Friedrich-Alexander University Erlangen-Nürnberg (FAU), Nägelsbachstraße 49a, 91052 Erlangen, Germany. Email: andre.pittig@fau.de

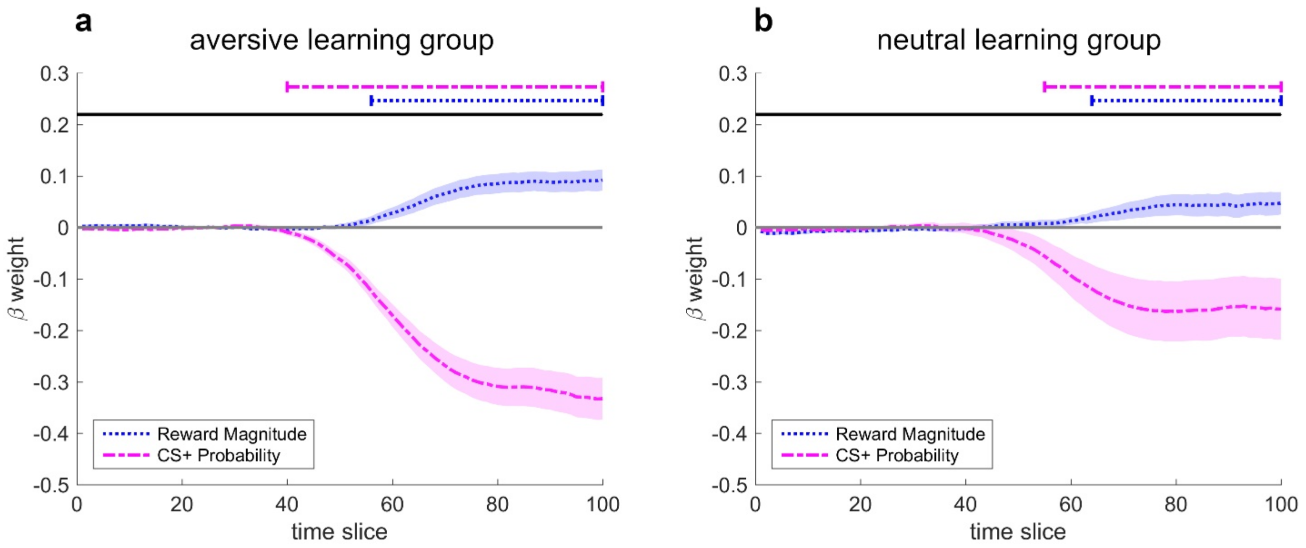

Supplementary Figure S1. Time-continuous beta weights from TCMR analysis in (a) the aversive learning group and (b) the neutral learning group for the predictors Reward Magnitude and CS+ Probability when excluding participants with a proportion of CS+/high reward choices > 90% or < 10%. Positive β weights indicate movement tendencies towards the CS+/high reward option, negative β weights indicate tendencies towards the CS-/low reward option. Horizontal lines at the top indicate segments of significant impact. Shaded areas represent standard error of the mean. Only segments of more than 10 successive significant t-tests were accepted as meaningful. In the aversive learning group, a time slice corresponds on average to 7.8 ms. In the neutral learning group, a time slice corresponds on average to 7.7 ms.

Supplementary Table S1. Segments of significant beta weights for Reward Magnitude and CS+ Probability separately for the aversive learning group and the neutral learning group when excluding participants with a proportion of CS+/high reward choices > 90% or < 10%.

	**aversive learning group**				**neutral learning group**			
Beta for	Start	End	Duration	Peak strength	Start	End	Duration	Peak strength
Reward Magnitude	56	100	44 (≈ 343.2 ms)	.092	64	100	36 (≈ 277.2 ms)	.047
CS+ Probability	40	100	60 (≈ 468.0 ms)	.334	55	100	45 (≈ 346.5 ms)	.164

Note. In the aversive learning group, a time slice corresponds on average to 7.8 ms. In the neutral learning group, a time slice corresponds on average to 7.7 ms. Only segments of more than 10 significant *t*-tests were accepted as meaningful.

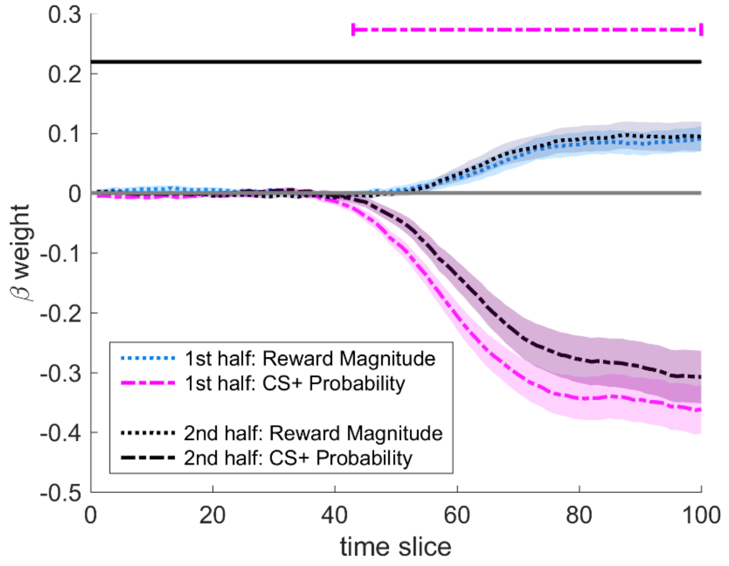

Supplementary Figure S2. Time-continuous beta weights from TCMR analysis for the predictors Reward Magnitude and CS+ Probability for the first half (light-colored) and the second half (dark-colored) of the approach-avoidance paradigm in the aversive learning group when excluding participants with a proportion of CS+/high reward choices > 90% or < 10%. Positive β weights indicate movement tendencies towards the CS+/high reward option, negative β weights indicate tendencies towards the CS-/low reward option. Horizontal lines at the top indicate segments of significant difference between both halves for a specific predictor. Shaded areas represent standard error of the mean. Only segments of more than 10 successive significant t-tests were accepted as meaningful. In the aversive learning group, a time slice corresponds on average to 7.8 ms.

Supplementary Table S2. Segments of significant beta weights for Reward Magnitude and CS+ Probability separately for the first and the second half of the approach-avoidance paradigm in the aversive learning group when excluding participants with a proportion of CS+/high reward choices > 90% or < 10%.

Beta for	**first half**				**second half**				**first vs. second half**		
	Start	End	Duration	Peak strength	Start	End	Duration	Peak strength	Start	End	Duration
Reward Magnitude	58	100	42 (≈ 327.6 ms)	.091	56	100	44 (≈ 343.2 ms)	.097	-	-	-
CS+ Probability	39	100	61 (≈ 475.8 ms)	.363	46	100	54 (≈ 421.2 ms)	.308	43	100	57 (≈ 444.6 ms)

Note. In the aversive learning group, a time slice corresponds on average to 7.8 ms. Only segments of more than 10 significant *t*-tests were accepted as meaningful.

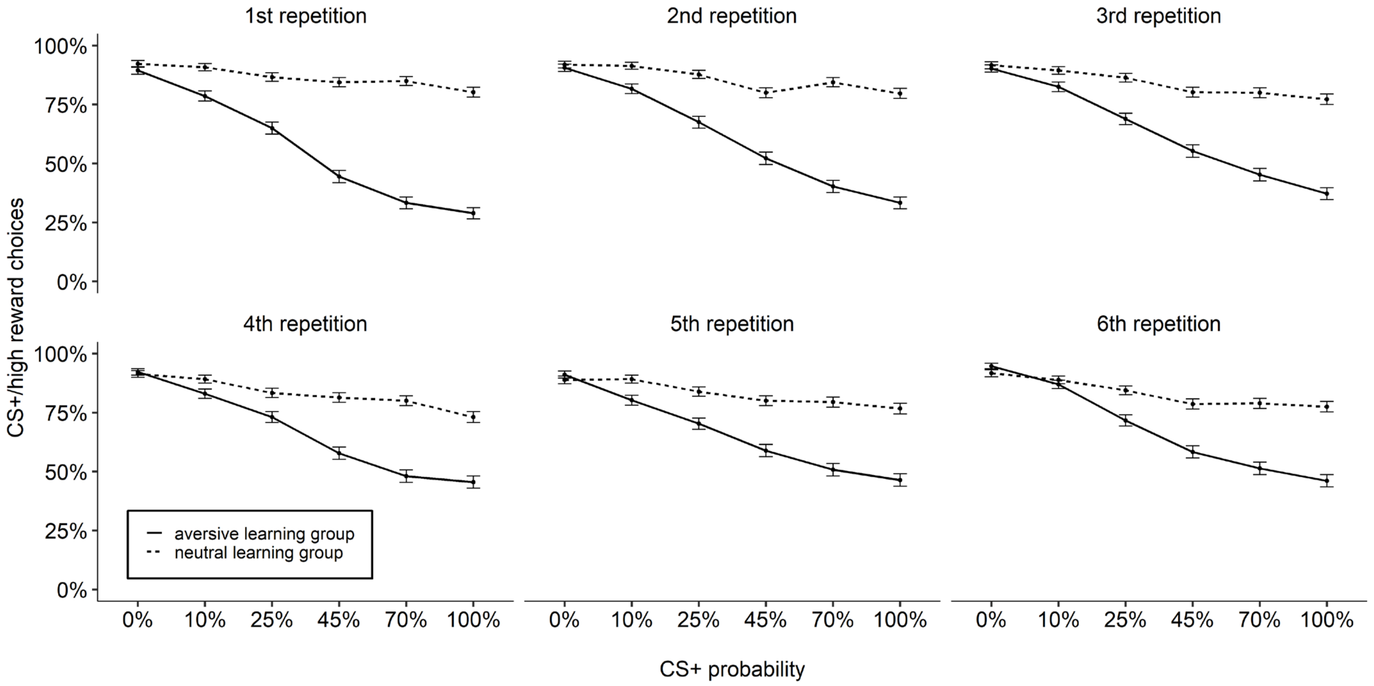

Supplementary Figure S3. Mean percentage of CS+/high reward choices (with standard error) in relation to CS+ probability, separately for each trial repetition, i.e., the number of times a specific trial had been presented during the task (1st to 6th repetition).

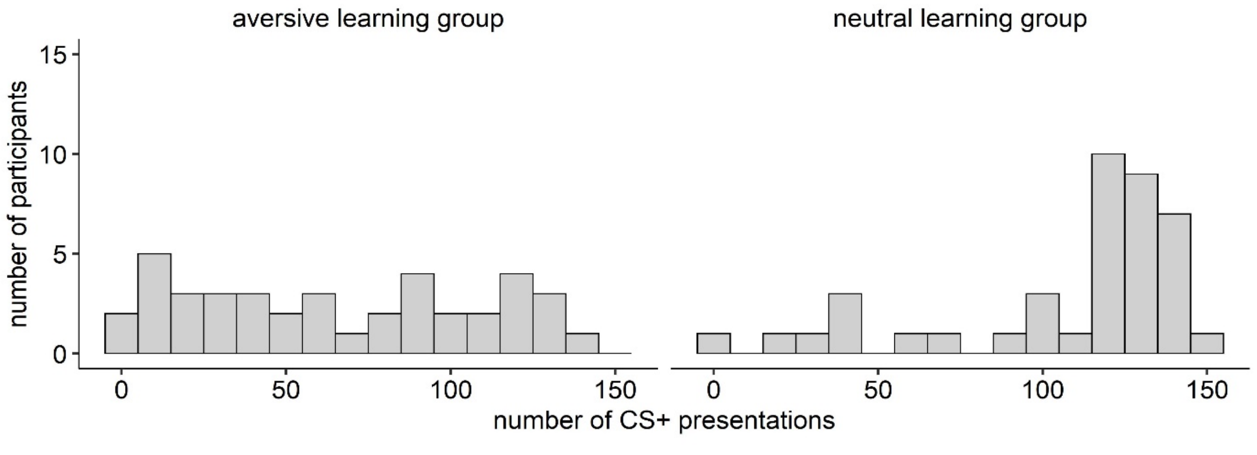

Supplementary Figure S4. Total number of CS+ presentations during the approach-avoidance paradigm for each participant of the aversive learning group and the neutral learning group.
